# The Hammerhead Ribozyme: A Long History for a Short RNA

**DOI:** 10.3390/molecules22010078

**Published:** 2017-01-04

**Authors:** Marcos de la Peña, Inmaculada García-Robles, Amelia Cervera

**Affiliations:** 1 Instituto de Biología Molecular y Celular de Plantas (IBMCP) (CSIC-UPV), C/Ingeniero Fausto Elio s/n, 46022 Valencia, Spain; amcerol@ibmcp.upv.es; 2 Department of Genetics, University of Valencia, C/Dr. Moliner 50, Burjassot, 46100 Valencia, Spain; garciai@uv.es

**Keywords:** phosphodiester bond, RNA catalysis, self-cleaving

## Abstract

Small nucleolytic ribozymes are a family of naturally occurring RNA motifs that catalyse a self-transesterification reaction in a highly sequence-specific manner. The hammerhead ribozyme was the first reported and the most extensively studied member of this family. However, and despite intense biochemical and structural research for three decades since its discovery, the history of this model ribozyme seems to be far from finished. The hammerhead ribozyme has been regarded as a biological oddity typical of small circular RNA pathogens of plants. More recently, numerous and new variations of this ribozyme have been found to inhabit the genomes of organisms from all life kingdoms, although their precise biological functions are not yet well understood.

## 1. Introduction

**Figure 1:**
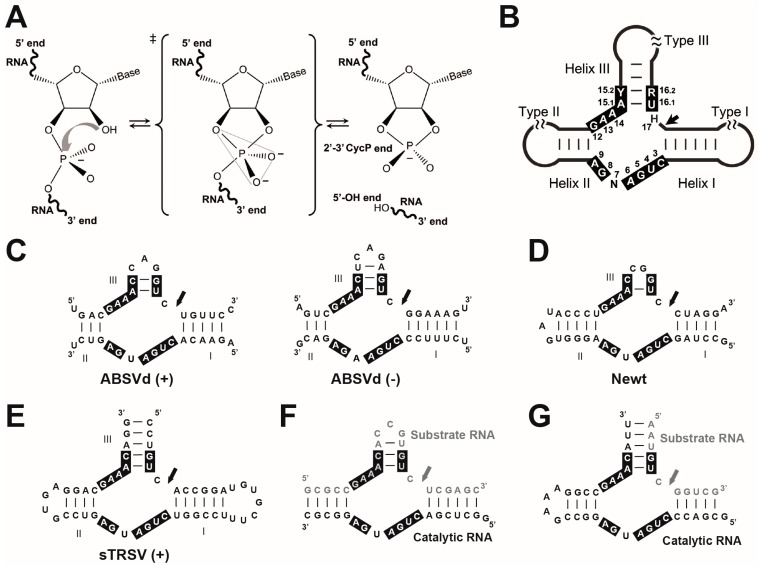
(**A**) Mechanism of internal transesterification reaction in the RNA. The cleavage reaction proceeds with an attack of the hydroxyl moiety at 2′ to the phosphate group at 3′, followed by a bipyramidal transition-state. The cleavage products are a 2′-3′-cyclic phosphate at the 5′ RNA product and a 5′-hydroxyl at the 3′ RNA product; (**B**) Diagram of the hammerhead ribozyme. Black boxes indicate the highly conserved nucleotides at the catalytic core. Secondary structures of (**C**) the HHRs found in ASBVd; (**D**) newt genome; and (**E**) sTRSV, as well as (**F**) the first reported HHR acting in *trans*; and (**G**) a more typical *trans*-acting HHR construct based on type III motifs. The sites of self-cleavage are indicated by arrows.

RNA catalysis in biology is mostly considered as an unusual feature of the RNA molecule. The first demonstration that polypeptides can be biological catalysts was published as early as 1926 [1], and consequently, the discovery in the 80s of catalytic RNAs or ribozymes was a really late paradigm change. In 1982, the self-splicing Group I intron was reported as the first discovered catalytic RNA [2]. It was described in the ciliate protozoa *Tetrahymena thermofila*, although similar introns can be found in some prokaryotic genomes and the mitochondria and chloroplast DNA of diverse eukaryotes. The second example of a ribozyme to be discovered was the RNAse P involved in tRNA maturation, which had a key biological role and a ubiquitous occurrence [3]. Interestingly, and despite the bacterial origin of the chloroplast and mitochondria, the RNAse P in these organelles is a protein that functions without RNA [4,5]. The third reported catalytic RNA was a tiny ribozyme (~50 nt), the self-cleaving hammerhead ribozyme (HHR), which was found in a group of atypical plant pathogens with small circular RNA (circRNA) genomes such as viral satellite RNAs [6] and viroids [7]. Since then, a few more examples of either natural or artificial ribozymes have been discovered, including the ribosome, a singular catalytic RNA that catalyzes the peptide bond formation, the central chemical reaction in extant biology [8]. This landscape strongly supports the hypothesis of a prebiotic RNA world, where the first self-replicating organisms were based on RNA as both the genetic material and as catalyst [9]. Whereas modern proteins would have replaced most of these ancient catalytic RNAs, some of them have remained in current organisms performing different functions. Among all known ribozymes, there is the enigmatic family of small (<200 nt) self-cleaving RNAs, which catalyse a simple intramolecular transesterification in a highly sequence-specific manner. This reaction, which can occur spontaneously in the RNA, starts by a S_N_2-like nucleophilic attack of the 2′-oxygen to the adjacent 3′-phosphate, resulting in cleavage of the phosphodiester bond to form a 2′-3′-cyclic phosphate and a 5′-hydroxyl RNA products (Figure 1A). Similarly to ribonuclease proteins such as the RNAse A, small self-cleaving ribozymes stabilize the formation of the bipyramidal oxyphosphorane transition-state through different catalytic strategies, such as in-line atomic orientation, electrostatic neutralization, general base and general acid catalysis [10]. In this way, nucleolytic ribozymes are able to catalyze RNA cleavage at a rate only a few-fold slower than their protein counterparts [11,12,13], which are thought to enhance the uncatalyzed rate of unspecific cleavage about 10^11^-fold [14]. At least nine classes of naturally-occurring small self-cleaving ribozymes have been described so far: the hammerhead [6,7], hairpin [15], human Hepatitis-δ [16], Varkud-satellite [17], GlmS [18], twister [19], twister sister, hatchet and pistol [20] ribozymes. Since its discovery 30 years ago, the HHR has been extensively used as a model ribozyme for structural, biochemical and biological studies. It is composed of a catalytic centre comprising 15 highly conserved nucleotides surrounded by three double helixes (I to III), which adopt a secondary structure that resembles the shape of a hammerhead shark head. Depending on the open-ended helix, there are three possible circularly permuted forms, named type I, II or III (Figure 1B). The HHR motif, like other small ribozymes such as hairpin and Hepatitis-δ, has been historically regarded as a biological peculiarity of subviral circRNA genomes [21]. However, we know now that small catalytic RNAs such as the HHR can occur numerously in DNA genomes from bacteria to eukaryotes, including our own genome, and carrying out diverse biological functions that we are just starting to learn.

## 2. The Discovery of the HHR in Infectious circRNAs of Plants

An atypical group of autonomous plant pathogens composed of a small circRNA were reported in the 70s and called viroids [22]. Similar infectious circRNAs, named viroid-like satellite RNAs, were also known to occur associated with some plant RNA viruses [23]. In 1986, RNA self-cleaving activity was independently reported for the satellite RNA of the Tobacco Ringspot virus (sTRSV) [6] and the Avocado Sunblotch viroid (ASBVd) [7]. Based on the ASBVd sequence, comparison studies allowed to propose the first model for the hammerhead fold, which in the case of this viroid can be regarded as a kind of type I HHRs with a relatively unstable helix III, especially in the case of the positive polarity (Figure 1C) [7]. Curiously, ASBVd HHRs are among the most atypical motifs known for this ribozyme, with no clearly delimited helix I and II. Soon after these discoveries, self-cleaving activity due to a type I HHR was reported in a radically different environment: the so-called satellite DNA (repetitions of a DNA sequence of a few hundred bp) of newt genomes (Figure 1D) [24]. Biochemical analysis of both viroid and newt motifs revealed a dimeric mechanism for self-cleavage catalysis, where two tandem copies of the HHR in the same RNA molecule adopt a conformation with an elongated helix III that permits much higher self-cleaving efficiency in vitro than the monomeric form of the ribozyme [25]. The type III HHR fold (Figure 1E), on the other hand, was defined by analysis of diverse satellite RNAs. In contrast with the observed behaviour for type I motifs, type III HHRs self-cleave in vitro with high efficiency as monomers [26,27]. Since then, the field of small circRNA replicons of plants and the continuous discovery of newer members have offered a collection of more than 20 different examples of HHRs, many of them with their own structural and biochemical peculiarities [28,29,30].

## 3. *trans* RNA Cleavage Using a Minimal HHR Motif: Too Minimal as a Model

In natural conditions, HHR and most nucleolytic ribozymes are known to act exclusively in *cis*, carrying out the self-cleavage of the RNA molecule. Formally, this cleavage reaction cannot be considered truly catalysed due to the consumption during the reaction of the catalyst. However, soon after the HHR discovery, Uhlenbeck noticed that naturally-occurring HHRs can be split in two RNA pieces: one oligoribonucleotide acts as a true catalyst over different rounds of cleavage reaction in *trans* on a second specific oligoribonucleotide substrate [31]. To perform these studies, the analysed HHR was an artificial variant based on the ASBVd and newt HHRs, which lacked of any loop sequences at the helix I or II (Figure 1F). These loops present in naturally-occurring HHRs, however, were found afterwards to be crucial for the understanding of the real catalytic mechanism of this ribozyme (see below). Nevertheless, these first kinetic studies worked reasonably well, although under non-physiological conditions (i.e., high Mg^2+^ concentration). On the other hand, most of the HHR constructs designed to act in *trans* for either basic or applied research were based on the type III motifs lacking any loop at helix I (Figure 1G).

**Figure 2:**
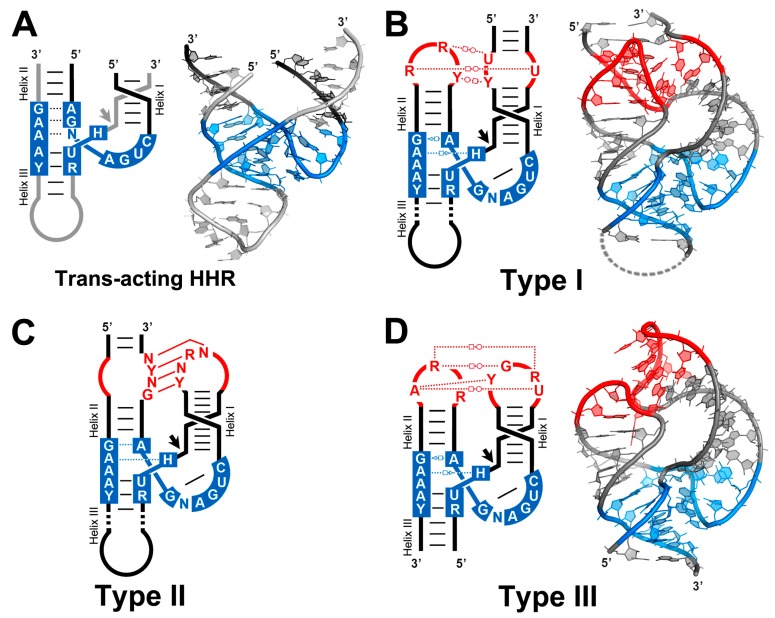
(**A**) Schematic 3D representation (**left**) of an artificial minimal hammerhead with the ribozyme strand (in black) acting in *trans* on the substrate strand (in grey), and a crystallographic model (**right**) of a trans-acting HHR in the inactive conformation; (**B**) Schematic 3D representation of a type I HHR (**left**) and its crystallographic model based on the *S. mansoni* HHR (**right**). A loop of helix III not included in the crystallized RNA is drawn with a dotted grey line; (**C**) Schematic 3D representation of a typical type II HHR present in prokaryotic genomes. Tertiary interactions in type II are usually Watson-Crick base pairs. No crystallographic models for any of these HHRs are available for the moment; (**D**) Schematic 3D representation of a type III HHR (**left**) and the crystallographic model based on the sTRSV HHR (**right**). The sites of self-cleavage are indicated by arrows. The highly conserved nucleotides of the core are shown in blue, whereas interacting loops appear in red. Watson-Crick interactions are indicated with solid lines, whereas dotted lines indicate noncanonical base pairs, including the symbols previously proposed for the specific hydrogen bonding interactions [38].

The self-cleaving motif of sTRSV was not only the first discovered HHR, but also the first catalytic RNA to provide high resolution crystals [32], although this structure was not solved until 20 years later (see below). In contrast, artificial *trans*-acting HHRs derived from the biochemical studies mentioned above were crystallized and solved structurally, first as a DNA-RNA hybrid [33] and then as a full RNA-RNA complex (Figure 2A) [34]. Both 3D models similarly showed that these HHRs fold into a γ-shaped three-way junction comprising a near-collinear stacking of stems III and II, which is packed next to stem I thanks to a classical uridine turn structure [35,36]. The structures revealed that the catalytic core of the ribozyme was probably trapped in a pre-catalytic state, suggesting that the RNA would require important rearrangements to bring the key nucleotides into position for in-line attack. In consequence, most of the biochemical and structural data published for the HHR conflicted for a decade, until the structure of a full natural HHR came to light. During this time full of contradictions, the HHR was mostly considered a metalloenzyme, where divalent cations such as Mg^2+^ would be the acid-base catalytic components. However, the discovery that this and other small self-cleaving RNAs such as the Hepatitis-δ or Varkud ribozymes were catalytically active under high concentrations of non-metallic monovalent ions indicated that RNA alone would be sufficient for self-cleaving catalysis [37].

## 4. Tertiary Interactions of the HHR Allow for a More Efficient Self-Cleavage In Vivo

Since its discovery, minimal versions of the HHR lacking peripheral loops were mostly used by the scientific community to study this ribozyme. As originally pointed out by McKay, the existence of controversial issues in the area indicated that the history of the HHR was far from finished [39]. In this regard, missing parts were already advanced by pioneer work with the type III HHR of the satellite RNA of the Lucerne Transient Streak virus (sLTSV), which revealed that self-cleavage of the purified RNA was quantitative within 1 min, impeding determination of the rate of cleavage [27]. In this line, initial studies with type I HHRs from newts and salamanders showed that self-cleaving catalysis was also possible for single monomeric motifs [40], which required internally looped extensions of helix I [41] only compatible with specific loops at helix II [42]. Other work done with naturally-occurring HHR motifs such as the ribozyme of satellite RNA of Cereal Yellow Dwarf virus-RPV (sCYDV-RPV, formerly known as Barley yellow dwarf virus satellite RNA) indicated that loop interactions between helix I and II somehow controlled self-cleavage catalysis [43]. It was not until 2003 that two independent publications concluded that loop-loop interactions between these two helixes were required to reach high activity under the low magnesium concentration found in vivo [44,45]. The work of both groups revealed that naturally-occurring type III HHRs keeping loops 1 and 2 dramatically increased their observed catalytic rate of cleavage (>100 min^−1^) in comparison with the same versions lacking one of the loops (~1 min^−1^). Moreover, changes in the loop sequences induced a large reduction in the cleavage rate (<0.01 min^−1^) [44], suggesting that steric clashes prevented the necessary and specific interactions for proper folding of the ribozyme. Similar results were obtained for other HHRs, including the type I HHR encoded in the satellite DNA Smα of the *Schistosoma mansoni* trematode [46], which reached a maximum cleavage rate close to 1000 min^−1^ [10]. Moreover, detailed kinetic analysis of the full *S. mansoni* HHR also revealed a 2000-fold increase in the rate of ligation compared to minimal hammerheads without tertiary interactions [47].

## 5. The Structure of a Full HHR

**Figure 3:**
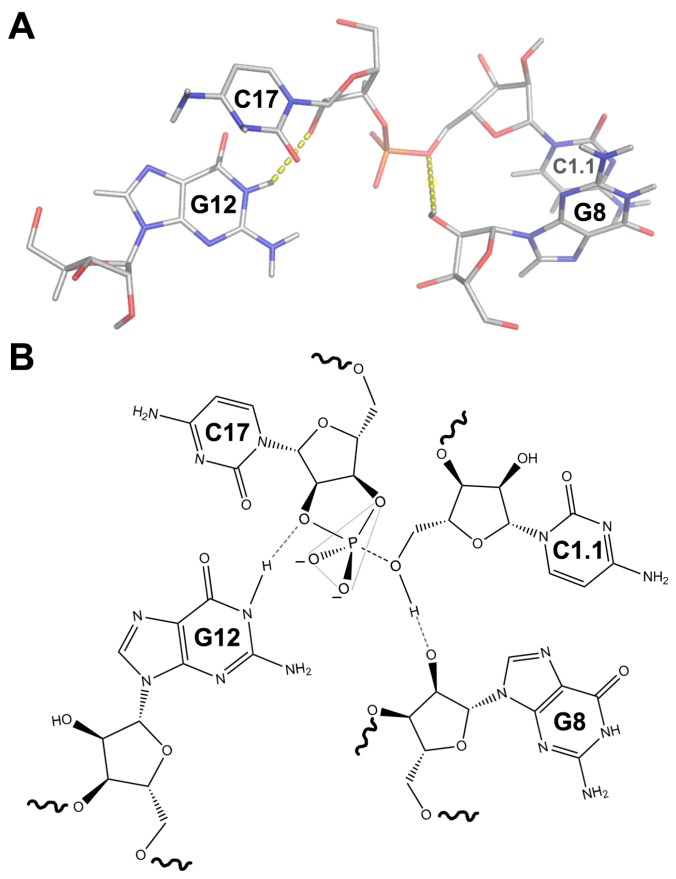
(**A**) Close-up view of the catalytic centre of the *S. mansoni* HHR; (**B**) Schematic representation of the structure shown in (**A**) including the proposed mechanism of catalysis and the formation of the transition state.

The Scott group solved in 2006 the structure of a full HHR of *S. mansoni* (Figure 2B), which clearly revealed how tertiary interactions in the peripheral regions of the RNA prime the ribozyme for catalysis [48]. As observed for the minimal HHR motif, the full ribozyme has a similar γ-shaped fold, but with a totally rearranged catalytic centre (Figure 2B) where we find the 2′-O nucleophile properly aligned with the scissile phosphate in a structure compatible with a general acid-base mechanism of catalysis. Such a stabilization of the precatalytic structure in the full but not in the minimal ribozymes is believed to accelerate the self-cleavage reaction. A detailed view of the rearranged core shows that the G12 residue is acting as the general base in the reaction that might deprotonate the 2′-OH of the residue at position 17 to generate the attacking nucleophile. On the other hand, the general acid may be represented by the 2′-OH of G8 that interacts with the leaving oxygen (Figure 3). Altogether, these new interactions in the catalytic core and the proposed mechanism of acid–base catalysis allowed to explain most of the biochemical discrepancies in the field. An equivalent key role of the peripheral regions of the HHR in the conformation of the active site and in catalysis has been observed in other macromolecules such as protein enzymes and other ribozymes. In those cases, a properly packed global structure provides molecular rigidity allowing maximal stabilization of the transition-state relative to the ground state, and therefore maximizing catalysis [49,50].

Following the structure determination of the *S. mansoni* type I HHR, new structural models for type III HHRs were also published [51,52]. The catalytic center of the type III HHR of sTRSV was almost identical to the one reported for the *S. mansoni* motif, which confirmed the proposed mechanism of catalysis [51]. A close-up view of the loop-loop interactions showed that they all take place across the major groove of the RNA helixes and comprise a network of non-canonical base pairs and interdigitations (Figure 2B,D). Despite the different sequences and topologies naturally found in loops of helixes I and II, a conserved reverse Hoogsteen pair seems to occur in both type I and III HHRs [52,53]. A second conserved interaction in most type III motifs is a U:A:U base triple, whereas Type I motifs conserve a second reverse Hoogsteen and a Watson-Crick/Hoogsteen pairs (Figure 2).

## 6. Widespread Occurrence of the HHR along the Tree of Life

Since the discovery of the HHR, the occurrence of these catalytic motifs in DNA and RNA genomes offered a really puzzling panorama. The initial discovery of the HHR in plant pathogenic circRNAs somehow pigeonholed this and other self-cleaving RNAs, such as the Hepatitis-δ and hairpin ribozymes, into the world of subviral agents (see before). Moreover, RNA self-cleavage had a clear biological role in the replication process of these circRNAs through a classical rolling-circle mechanism [54]. The HHRs found in the satellite DNA of newts and salamanders [24,55] however, were an unexpected discovery, for which a biological role was unknown [56]. In the following years, a few more unexpected examples of eukaryotic HHRs were described in the genomes of two plants, carnation [57] and *Arabidopsis thaliana* [58], and two invertebrates, *S. mansoni* [46] and cave crickets [59]. The HHRs found in invertebrates and newts showed some shared characteristics, occurring in both cases as close tandem copies associated with repetitive DNA. Previous bioinformatic analyses suggested that a few hundred HHR motifs could be found in the genomic databases [60], although these searches were performed using minimal motifs with a quite relaxed sequence at the catalytic centre and, consequently, most of the reported motifs were likely false positives. With the discovery of two canonical type III HHRs in the genome of *A. thaliana* [58], the labs of Hammann and Westhof suggested that we were far from knowing the full spectrum of the diversity of catalytic RNAs, and deeper bioinformatic analyses could be the key to solve this question [61]. In this line, all the knowledge about conserved tertiary interactions in the HHR combined with simple homology searches was initially used in our lab to perform bioinformatic searches in the genomic databases. In this way, we revealed examples of canonical type I and type III HHRs in intergenic regions of several bacterial genomes as well as in metagenomic data with very different origins [62]. These results indicated that not only genomes from the subviral and eukaryotic domains but also prokaryotes contained genomic HHRs. Many more examples of prokaryotic HHRs, including cases in archaeal genomes, were also reported independently by the Breaker and Lupták labs, which also discovered the existence of type II HHRs, a totally new topology for this ribozyme (Figure 2C) [63,64]. In many instances, different HHR motifs were found close to each other and flanking ORFs encoding small and non-conserved proteins with unknown functions. Moreover, comparative genomic analyses have recently allowed the discovery of new self-cleaving motifs associated with many of these ORFs [20], suggesting that this simple catalytic activity could be performed by many different structures. Although the possible biological roles for prokaryotic HHRs and related self-cleaving motifs remain one of the open questions in the field, many instances suggest a persistent relationship with bacteriophage genomes, either free-living or as integrated sequences in bacterial genomes (i.e., prophages) ([19,20,62,63,64]; De la Peña, unpublished results).

**Figure 4:**
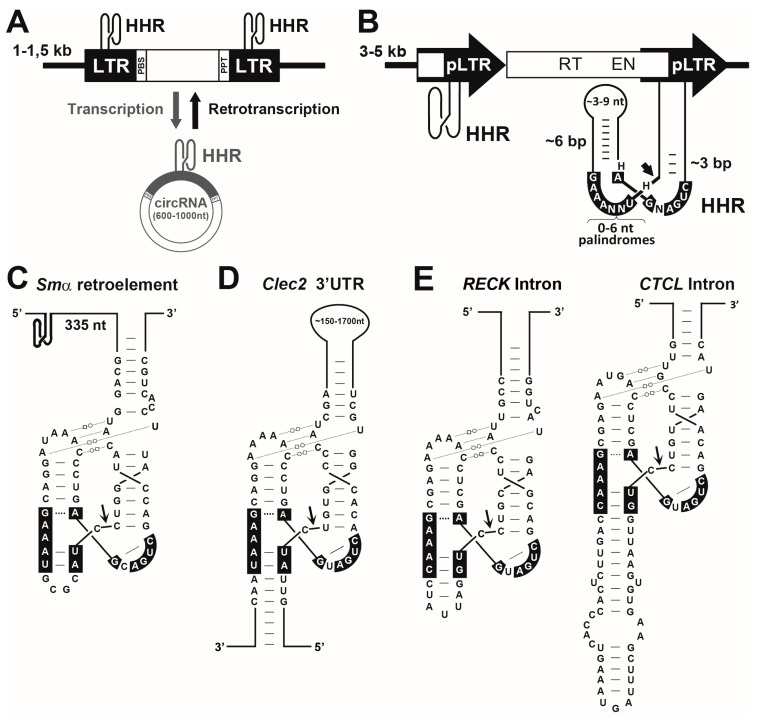
(**A**) Diagram of a genomic retrozyme harbouring a type III hammerhead ribozyme (HHR) in each Long Terminal Repeat (LTR). After transcription of the retrozyme, double self-cleavage and circularization, a circular RNA (circRNA) is generated. The circRNA in turn can be retrotranscribed and integrated as a new retrozyme in the plant genome; (**B**) Diagram of a Penelope-like retroelement (PLE) containing minimal hammerhead ribozymes in their LTRs; (**C**) Type I hammerhead ribozymes detected in Smα, a non-autonomous retrotransposon of the trematode *S. mansoni*; (**D**) The first mammalian hammerhead ribozyme detected in the 3′ UTR of *Clec2* genes of rodents; (**E**) The first human hammerhead ribozymes detected in the introns of the *RECK* (**left**) and *CTCL* (**right**) genes. Conserved tertiary interactions are indicated with dotted lines and the symbols previously proposed for the specific hydrogen bonding interactions [38]. The sites of self-cleavage are indicated by arrows.

Regarding the widespread occurrence of HHRs in eukaryotic genomes, bioinformatic searches revealed numerous new examples of classic type I HHRs in the satellite DNAs of either unicellular (i.e., protists) or multicellular organisms (mostly metazoans, from cnidarians to lower vertebrates) [62,64,65,66], as well as canonical type III HHRs in plant genomes, which usually appear as tandem repeat copies [62]. Deeper analysis of the genomic HHRs in plants showed that they are part of a new family of mobile genetic elements named retrozymes (after non-autonomous retroelements with hammerhead ribozymes) [67]. Interestingly, retrozymes seem to spread in plant genomes through small (600–1000 nt) non-coding circRNAs (Figure 4A), which directly connect with the circRNA replicons where the HHR was originally discovered ([67]; De la Peña and Cervera, in press). A further connection between HHRs and retrotransposons has been discovered in the ancient family of Penelope-like elements (PLEs), which are present in genomes from lower eukaryotes to many invertebrates and lower vertebrates [68,69]. Bioinformatic analyses showed that all available PLE sequences display the conserved presence of HHR motifs, which usually correspond to minimal versions of this ribozyme lacking the helix III and the characteristic tertiary interactions (Figure 4B).

## 7. RNA Self-Cleavage Catalysis in Biology: From Mobile Genetic Elements to Domesticated New Functions

Most of the current data on the biology of the genomic HHRs point to a common role of this ribozyme in the propagation of diverse retrotransposons. This view is in agreement with similar results observed for other self-cleaving RNAs such as Group I/II introns [2], Hepatitis-δ [70,71] or Varkud [72] ribozymes among others. Mobile genetic elements such as retrotransposons are major components of eukaryotic genomes, which have been historically regarded as junk DNA. More recent data, however, indicate that mobile genetic elements, besides being genomic parasites, are major players in genome evolution responsible for the development of many aspects of eukaryotic complexity. Molecular domestication would be just a consequence of the main idea that evolution co-opts what is already present in the genomes as the building blocks for novel molecular systems. In this regard, some HHRs of retroelements in lower eukaryotes (Figure 4C) seem to have been domesticated in the genomes of complex animals, such as reptiles, birds and mammals, to perform new biological functions. In 2008, the first example of a likely co-opted HHR in mammalian genomes was found in the 3′ UTR of the *Clec2* genes of different mammals, including some rodents and platypus, but not humans [73,74]. This self-cleaving motif was a very atypical type III HHR, the so called discontinuous HHR, characterized by a very large helix I (from 150 to 1500 nt, depending on the mammalian species) (Figure 4D). Although the biological function of this self-cleaving motif is still unknown, a role in the control of the site of polyadenylation could be suggested. In 2010, very similar type I HHRs were found conserved in the introns of some genes of all amniotes analysed (reptiles, birds and mammals) (Figure 4E) [66]. As the most striking case, one of these intronic HHRs was found totally conserved in the *RECK* gene of all warm-blooded animals (birds and mammals), whereas some other HHR motifs mapped in the introns of the *CTCL* gene of most mammals or the *DTNB* gene of birds and reptiles, suggesting a role in the biogenesis of the mRNAs harbouring these ribozymes [66,75]. The high sequence and structural similarity between intronic HHRs in amniotes and those found in the retroelements of diverse metazoans from trematodes to lower vertebrates, such as coelacanth fishes or amphibians, suggests that these ribozymes would have been domesticated during evolution to perform new and conserved functions in complex metazoans such as amniotes [66]. As a feasible hypothesis, highly conserved HHRs in the introns of diverse genes of amniotes could represent a new form or regulation of the alternative splicing, which in some cases may result in the production of crucial gene isoforms for these organisms [75]. Most likely, future bioinformatic analysis will reveal more examples of genomic HHRs conserved either in different non-coding regions, which will help us to better understand all the capabilities of these small ribozymes as gene regulatory elements.

## 8. Conclusions

The current era of genomics has opened many new doors in the biological knowledge, and one of these is the renaissance of the interest in self-cleaving ribozymes and RNA catalysis in general. The extreme simplicity and little sequence conservation of small ribozymes, together with the huge sequence space available for bioinformatic searches, make the identification of these small motifs a difficult task, usually hindered by a large collection of false positives, which require more detailed evolutionary and/or structural analyses to be filtered out. The recent discoveries of totally new self-cleaving motifs indicate that we could be just scratching the tip of an iceberg of naturally-occurring ribozymes, an iceberg that was first spotted with the discovery of the HHR.
